# Engineered mammalian and bacterial extracellular vesicles as promising nanocarriers for targeted therapy 

**DOI:** 10.20517/evcna.2022.04

**Published:** 2022-04-13

**Authors:** Han Liu, Zhen Geng, Jiacan Su

**Affiliations:** Institute of Translational Medicine, Shanghai University, Shanghai 200444, China.; ^#^Authors contributed equally.

**Keywords:** Biological engineering, chemical modification, mammalian extracellular vesicles, bacterial extracellular vesicles, targeted therapy

## Abstract

Extracellular vesicles (EVs), which are nanocarriers with phospholipid bilayer structures released by most cells, play a key role in regulating physiological and pathological processes. EVs have been investigated due to their loading capacity, low toxicity, immunogenicity, and biofunctions. Although EVs have shown good potential as therapeutic vehicles, natural EVs have a poor targeting ability, which substantially reduces the therapeutic effect. Through the addition of a targeting unit into the membrane surface of EVs or inside EVs by engineering technology, the therapeutic agent can accumulate in specific cells and tissues. Here, we focus on mammalian EVs (MEVs) and bacterial EVs (BEVs), which are the two most common types of EVs in the biomedical field. In this review, we describe engineered MEVs and BEVs as promising nanocarriers for targeted therapy and summarize the biogenesis, isolation, and characterization of MEVs and BEVs. We then describe engineering techniques for enhancement of the targeting ability of EVs. Moreover, we focus on the applications of engineered MEVs and BEVs in targeted therapy, including the treatment of cancer and brain and bone disease. We believe that this review will help improve the understanding of engineered MEVs and BEVs, thereby promoting their application and clinical translation.

## INTRODUCTION

According to the International Society for Extracellular Vesicles, extracellular vesicles (EVs) are defined as “particles naturally released from the cell that is delimited by a lipid bilayer and cannot replicate”^[1]^. Notably, the term “exosomes” is often used as a general description of EVs^[2,3]^. EVs can transmit a variety of biologically active components, such as proteins, nucleic acids, lipids, and metabolites, to affect the performance of recipient cells^[4-7]^. Many studies have found that EVs play an important role in regulating the physiological and pathological processes of the body by participating in cell-to-cell communication, cell proliferation, cell migration, angiogenesis, and immune regulation^[6,8]^. Due to their unique nanosized structures, loading capacity, biochemical properties, and good biocompatibility, EVs have been widely used in various applications in the biomedical field, such as vaccines, cancer agents, and drug delivery vehicles^[9-11]^. Although EVs have shown good potential as therapeutic vehicles, natural EVs were shown to have a poor targeting ability in animal experiments, which substantially reduced the therapeutic effect^[12,13]^. Therefore, many engineered methods have been applied to improve the targeting ability of EVs^[14-16]^.

In our previous study, we focused on engineering EVs derived from mammalian cells such as endothelial cells^[17] ^and NIH-3T3 cells^[18]^. Although these engineered mammalian EVs (MEVs) were shown to have an excellent targeting ability and therapeutic effect, the low extraction yield (requiring many mammalian cells) is still a limiting factor. The current complex and low yield protocols for purification and extraction of EVs, such as ultracentrifugation, gradient ultracentrifugation, co-precipitation, size-exclusion chromatography, and field flow fractionation, pose a tremendous challenge in the mass production of EVs. Therefore, we recently paid more attention to bacterial EVs (BEVs), which can be easily obtained through fed-batch fermentation and purification procedures^[4,13]^. Moreover, according to the latest minimal information for studies of extracellular vesicles (MISEV) in 2021, the topic of “nonmammalian EVs, especially BEVs” ranked fourth^[1]^. Although engineered BEVs have also been used in the field of biomedicine^[19,20]^, BEV research is less developed than that of MEVs. The number of MEV and BEV studies has increased rapidly in recent years (PubMed.gov). In general, MEVs and BEVs are the two most common types of EVs in the biomedical field.

Due to the importance of MEVs and BEVs in the field of biomedicine, we focus on these two types of EVs and their engineering and applications in this review. Here, to elucidate engineered MEVs and BEVs as promising nanocarriers for targeted therapy, we first summarize the biological basis of MEVs and BEVs, including different mechanisms of biogenesis, isolation, and characterization. We then present approaches for modifying BEVs and MEVs, which are physical engineering (membrane fusion and membrane coating), biological engineering [membrane fusion, lysosome-associated membrane glycoprotein 2B (LAMP-2B), and CD63], and chemical engineering (covalent reaction and noncovalent reaction**)**, to enhance the targeting ability. Finally, we conclude with the application of engineered MEVs and BEVs in targeted therapy of tumors (chemotherapy, gene therapy, photothermal therapy, and immunotherapy), brain disease [Alzheimer’s disease (AD), Parkinson’s disease (PD), and ischemic stroke], and bone disease [osteoarthritis (OA) and osteoporosis (OP)]. This review will help improve our understanding of the importance of MEVs and BEVs and thus promote targeted therapy for various diseases.

## THE BIOGENESIS OF MEVS AND BEVS

EVs are a general term for nanovesicles with phospholipid bilayer structures secreted by most cells^[2,3,21]^. EVs can be secreted by almost all cells and are widely present in cell supernatants and various body fluids^[22]^. As early as the 1960s, BEVs were first reported in the Gram-negative bacteria *Escherichia coli*^[23-26]^. In the 1980s, Pan and Harding *et al.*^[27,28]^successively observed the release of MEVs in reticulocytes. At this stage, both BEVs and MEVs were regarded as “garbage bags” for cells to discharge metabolic waste^[29]^. In 1996, Raposo *et al.*^[30]^ found that EVs derived from B lymphocytes can present antigens and activate T lymphocytes to participate in the regulation of immune cells. Soon after, EVs that could transfer nucleic acids such as mRNA and miRNA were also found in archaea^[31,32]^. Gradually, researchers discovered that the role of EVs is much more than clearance of cell waste; EVs also transmit signals to distant parts of the body, where they can affect multiple dimensions of cell life^[10]^. A detailed description of the mechanisms of MEVs and BEVs would provide an important theoretical basis for the treatment of disease. 

### The biogenesis of MEVs

**Figure 1:**
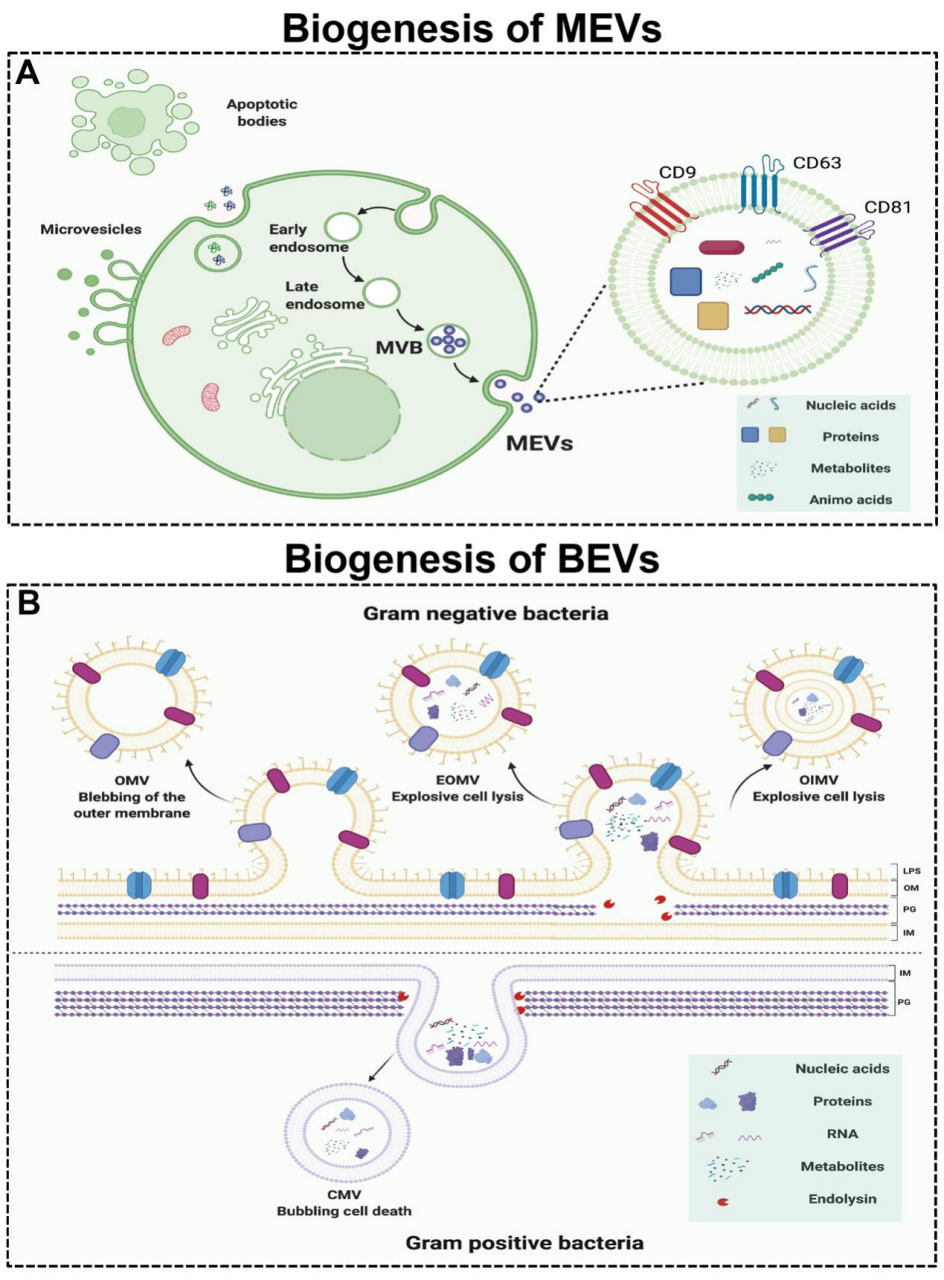
The biogenesis of mammalian extracellular vesicles (MEVs) and bacterial extracellular vesicles (BEVs). (A) The biogenesis of MEVs. (B) The biogenesis of BEVs. Figures were created with Biorender.com.

According to the size, biological characteristics, and formation process, MEVs can be classiﬁed into three major subtypes: exosomes, microvesicles, and apoptotic bodies [Figure 1A]^[33]^. Exosomes are EVs with a diameter of 40-160 nm formed by the fusion of multivesicular bodies (MVBs) and cell membranes. Moreover, microvesicles are EVs with a size range of 200-1000 nm in diameter that are directly formed by cell membrane budding. Apoptotic bodies are vesicular bodies with larger diameters (500-2000 nm in diameter) formed by cell fragmentation during the process of cell apoptosis. Among these subtypes, exosomes have received widespread attention due to their sizes, biological composition, and cell-to-cell communication ability^[10]^. Therefore, we use exosomes to represent MEVs in this review.

MEVs are formed by the endosomal system in a process involving three stages [Figure 1A]^[34]^. First, the plasma membrane invaginates to form endocytic vesicles, which fuse with each other to form early endosomes. Second, early endosomes invaginate again to encapsulate intracellular cargos, forming multiple intraluminal vesicles (ILVs), which are further transformed into late endosomes, MVBs. Finally, the MVBs fuse with the plasma membrane and excrete their contents into the extracellular space. The formation, sorting of cargos, and release of exosomes are a series of finely regulated processes that require the participation of many proteins. The formation of exosomes involves proteins such as endosomal sorting complex required for transport (ESCRT), transmembrane proteins (CD9, CD63, and CD81), apoptosis-linked gene 2-interacting protein X (Alix), and tumor susceptibility gene 101 protein (TSG101). Moreover, the intracellular transport of exosomes involves the participation of many molecular switches such as the RAB GTPase protein and cytoskeletal proteins such as actin and tubulin^[35]^. In addition, the secretion of exosomes requires the participation of SNARE protein complexes and the synaptic binding protein family^[36]^. The protein composition of exosomes can reflect the characteristics of their endosomal origin. In different types of cells and body fluids, exosomes all contain the same marker protein molecules, such as Alix, TSG101, SNARE, and RAB GTPase, and the transmembrane proteins, CD9, CD63, and CD81^[37]^. In addition to specific protein composition, exosomes also have a special lipid composition. Exosome membranes are enriched with cholesterol, ceramide, and sphingolipids^[38]^. These lipids are also involved in the formation and secretion of exosomes. For example, ceramide is involved in the budding of ILVs and MVBs^[38]^.

### The biogenesis of BEVs

Bacteria are divided into Gram-negative (G^-^) bacteria and Gram-positive (G^+^) bacteria based on their structure, morphology, and staining properties. Both G^+ ^and G^- ^bacteria can release EVs without energy consumption^[39,40]^. BEVs are EVs with sizes of 20-400 nm in diameter and can be divided into four types: outer-membrane vesicles (OMVs), explosive outer-membrane vesicles (EOMVs), outer-inner membrane vesicles (OIMVs), and cytoplasmic membrane vesicles (CMVs). The first three EVs are formed by G^- ^bacteria, and the latter are formed by G^+ ^bacteria. G^+ ^bacteria produce CMVs by endolysin-triggered cell lysis (bubbling cell death) [Figure 1B]^[26]^. OMVs are formed by blebbing of the outer membrane of G^- ^bacteria, while OIMVs and EOMVs are released by explosive cell lysis of G^- ^bacteria [Figure 1B]^[25]^. 

Similar to MEVs, BEVs are lipid bilayer-enclosed structures containing various biomolecules released by cells and are increasingly regarded as the main form of cell-to-cell communication^[41-43]^. Due to the diversity of their contents, BEVs have a key role in bacteria-bacteria and bacteria-host communications. Generally, BEVs contain high levels of proteins, nucleic acids, metabolites, small molecules, *etc. *G^- ^BEVs are enriched in periplasmic proteins such as the multidrug efflux pump subunit AcrA and outer membrane proteins such as outer membrane protein F (OmpF). Notably, Vanaja *et al.*^[44]^ used OmpF as a specific (surface) marker for *E. coli*-derived EVs. However, the lack of specific markers is still a challenge in the field of BEVs^[39]^. Moreover, lipids are an important structural component of bacterial cell membranes. The most significant difference between G^+ ^and G^- ^BEV contents is lipopolysaccharide (LPS, or endotoxin), which can cause an innate immune response^[45]^. Knockout of *msbB *in G^- ^bacteria *E. coli *for less endotoxic EVs is a common approach^[15,19,20,46]^. Interestingly, the nonpathogenic G^- ^bacteria *E. coli *Nissle 1917 lacks definite virulence factors such as LPS, so it can be used as a probiotic for the treatment of various gastrointestinal diseases^[47-49]^. Due to its easy genetic manipulation and probiotic characteristics, *E. coli *Nissle 1917 and its BEVs are promising candidates for medical engineering. In addition, BEVs have been reported to transfer nucleic acids, such as DNA and RNA, into other bacterial cells^[50] ^and mammalian cells, which trigger different host immune responses and cellular processes^[51,52]^. RNA, especially miRNA and siRNA, can be protected from degradation through BEVs, which promotes delivery to mammalian cells^[53]^. BEVs selectively package different metabolites depending on the strains. Gujrati *et al.*^[15] ^reported that the BEVs secreted by strains overexpressing melanin (Mel) also contain Mel. These findings indicate the applications of EVs in biomedicine.

## THE ISOLATION OF MEVS AND BEVS

**Figure 2:**
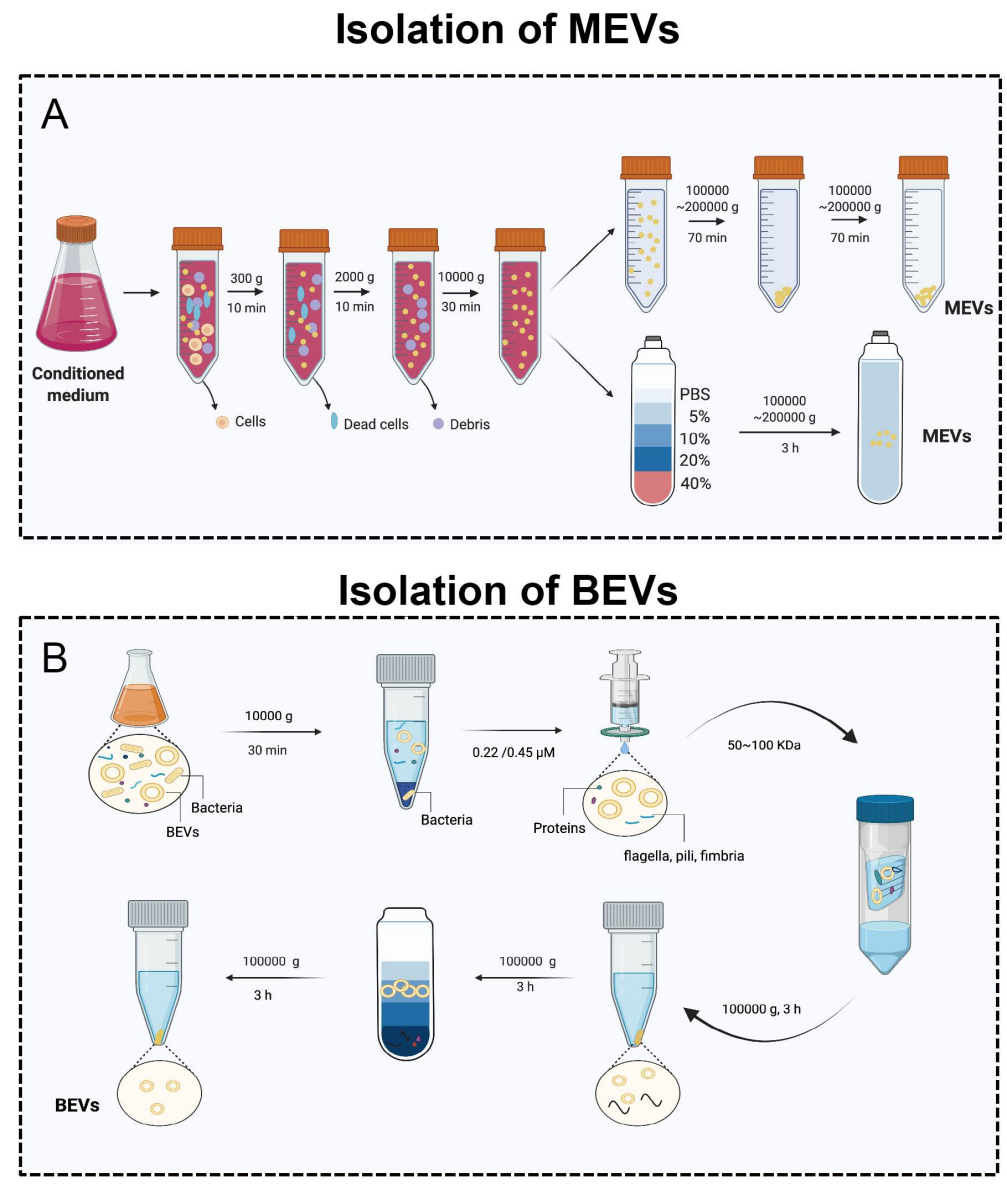
The isolation of MEVs and BEVs. (A) The isolation of MEVs. (B) The isolation of BEVs. Figures were created with Biorender.com.

The isolation and characterization of MEVs and BEVs is an indispensable step for their further application in biomedicine. In fact, the isolation of such nanoparticles is generally difficult. MEVs can be derived from a variety of biological fluids, such as blood serum^[54]^, breast milk^[55]^, urine^[56]^, tears^[57]^, saliva^[58]^, and sperm^[59]^. However, BEVs are found in many kinds of media, such as LB, MRS broth^[60]^, and BHI broth (which always requires porcine mucin for *Akkermansia muciniphila*)^[61]^. Therefore, there is a major difference in viscosity, which causes difficulties in isolation and purification. Moreover, the amount of sample available for isolation is another factor that affects efficiency. Recently, various isolation methods, such as differential centrifugation, precipitation, size exclusion chromatography, and magnetic capture, have been established based on the differences in size, density, charge, and surface ligands^[62-65]^. Here, we summarize the most commonly used and effective MEV and BEV isolation techniques [Figure 2].

### Isolation of MEVs

Ultracentrifugation-based MEV isolation is the gold standard, thus one of the most commonly used and reported techniques^[66-69]^. According to the first large-scale detailed survey of current global MEV isolation practices, 81% of researchers used ultracentrifugation (including differential centrifugation) for MEVs isolation^[68]^. Li *et al.*^[67] ^summarized the use of ultracentrifugation and differential centrifugation to remove other impurities in the sample through a combination of different speeds and times to finally achieve the isolation of MEVs. Low-speed centrifugation (300-2000 *g *or 2000-10,000 *g*) is generally used to remove cells, dead cells, cell debris, *etc. *Ultracentrifugation (100,000-200,000 *g*) is generally applied to collect MEVs [Figure 2B]. In addition, for better purification of MEVs, density gradient centrifugation such as iodixanol can be used [Figure 2A]. After the isolation of MEVs, the most commonly used characterization methods are transmission electron microscopy (TEM), nanoparticle tracking analysis (NTA), and Western blotting (WB)^[17,18,70,71]^. TEM and NTA are used to show the sizes, shapes, and concentrations of EVs. The transmembrane proteins CD9, CD63, and CD81, as well as TSG101, are often used as specific markers in WB^[17,18,70,71]^.

### Isolation of BEVs

The standard protocol for BEVs purification is to physically separate EVs from cell culture through a series of steps [Figure 2B]^[72-74]^. Simply, low-speed centrifugation (2000-10,000 *g*) is used to remove bacteria and their debris in the fermentation broth. Then, a 0.22 μm sterile filter is applied to remove residual bacteria. Subsequently, a 100 kDa ultrafiltration membrane is required to remove non-BEV-associated proteins. Furthermore, ultracentrifugation and density gradient centrifugation are used together for the separation and purification of BEVs. Using the above method, we successfully obtained multiple BEVs, such as EVs derived from *Lactobacillus rhamnosus *GG^[4]^. Similar to the characterization of MEVs, TEM and NTA are common methods used to assess BEVs. The outer membrane proteins OmpA^[20] ^and OmpF^[44] ^are used as specific markers for *E. coli*-derived EVs. However, many studies involving BEVs do not use WB^[15,19,75]^. The selection of specific markers is still a major challenge in the BEV field.

## ENGINEERING TECHNIQUES TO IMPROVE THE TARGETING ABILITY OF MEVS AND BEVS

**Figure 3:**
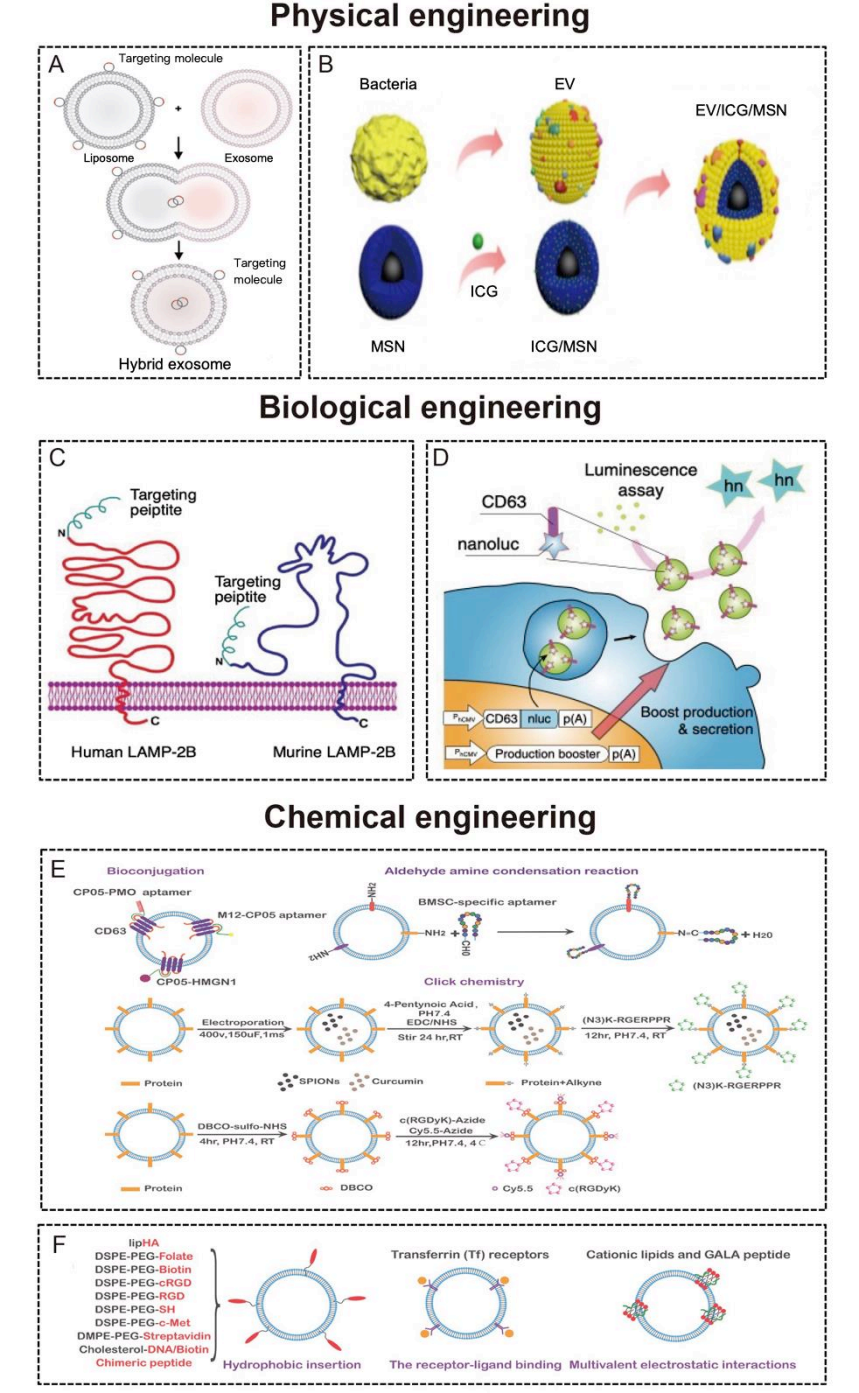
(A) The procedure to produce hybrid nanoparticles by membrane fusion. Liposomes with targeting molecules on the surface can be delivered into EVs through membrane fusion^[79]^. Copyright 2018 WILEY-VCH. (B) The procedure to produce hybrid nanoparticles by membrane coating^[80]^. Copyright 2020 Ivyspring International Publisher. (C) The fusion of the targeting peptide with LAMP-2B^[66]^. Copyright 2021 Ivyspring International Publisher. (D) The fusion of the delivery molecule with CD63^[81]^. Copyright 2018 Springer Nature. (E) Targeted modification of EVs based on chemical covalent reactions^[82]^. Copyright 2021 Elsevier. (F) Targeted modification of EVs based on chemical non-covalent reactions^[82]^. Copyright 2021 Elsevier. LAMP-2B: Lysosome-associated membrane glycoprotein 2B.

Nanosized EVs have been investigated as therapeutic vehicles due to their loading capacity, low toxicity, immunogenicity, and biofunctions^[10,76]^. However, the poor targeting ability of natural EVs is not conducive to therapy. With the introduction of the concept of precision medicine in 2015^[77]^, researchers are increasingly investigating the targeting ability of EVs. Targeted delivery could increase the local concentration of the therapeutic agent and minimize side effects. Through the addition of a targeting unit into the membrane surface of EVs or inside EVs by engineering technology, the therapeutic agent could accumulate in specific cells and tissues. Many engineering technologies such as biological engineering and chemical modification have been used to modify EVs to enhance their targeting ability. The membrane surface of MEVs is rich in lipoproteins (such as phosphatidylserine, cholesterol, sphingomyelin, and ceramide) and membrane proteins (adhesion molecules, integrins, membrane transport proteins, MHC class I/II, tetraspanins, and transferrin receptor)^[78]^. At present, the targeting engineering of MEVs is intensively studied^[13,16,66]^, but research on the targeting engineering of BEVs has just started^[15,20]^. Their similar phospholipid bilayer structure makes most engineering methods universal. Here, we summarize the techniques to improve the targeting ability of MEVs and BEVs [Figure 3 and Table 1].

**Table 1:** Summary of the techniques to improve the targeting ability of MEVs and BEVs

**Methods**	**Target cells/Tissue**	**Ref.**
**Physical engineering**		
Membrane fusion	Mesenchymal stem cells Bone mesenchymal stem cells	^[79]^ ^[18]^
Membrane coating	Lymph nodes	^[80]^
**Biological engineering**		
LAMP-2B	Neuronal cell Colorectal cancer (HCT-116) Synovial mesenchymal stem cells Chondrocyte	^[83]^ ^[84]^ ^[71]^ ^[85]^
CD63	Hepatocellular carcinoma (HepG2) CD8^+^ T-cells	^[86]^ ^[87]^
**Chemical engineering**		
Covalent reactions	Glioma Cerebral vascular endothelial cell	^[88]^ ^[12]^
Noncovalent reactions	Hepatoma 22 subcutaneous cancer cells Cancer cells (SKOV3, HCC-1954)	^[89,90]^ ^[20]^

MEVs: Mammalian extracellular vesicles; BEVs: bacterial extracellular vesicles.

### Physical engineering

Physical methods mainly include membrane fusion [Figure 3A] and membrane coating [Figure 3B]. The liposome mediated MEVs and BEVs membrane fusion strategy is an important engineering approach that endows EVs with specific functional ligands. Liposomes with targeting molecules on the surface can be delivered into EVs through membrane fusion. A mixture of MEVs and liposomes incubated at 37 °C for 12 h could form hybrid nanocarriers. Lin *et al.*^[79] ^developed MEV-liposome hybrid nanoparticles to accurately deliver large plasmids, such as CRISPR-Cas9, into mesenchymal stem cells (MSCs). Similarly, our team constructed MEV-liposome hybrid nanoparticles with the ability to target bone through spontaneous membrane fusion^[18]^. Yang *et al.*^[91] ^reported that virus-mimetic fusogenic MEVs could deliver membrane proteins to the target cell membrane by membrane fusion. Gao *et al.*^[92] ^also developed a virus-mimicking fusogenic vesicle with fusogenic proteins that could target sialic-acid-containing receptors on MEVs and promote membrane fusion. In addition, the fusion of MEVs derived from different cells and functionalized liposomes could be triggered by polyethylene glycol (PEG)^[93]^. 

On the other hand, membrane coating is a promising nanotechnology for disease-relevant targeting. The biological characteristics of cell membranes endow nanoparticles with broader applications, including targeting ability^[94]^. Various cell types, including mammalian cells (such as red blood cells^[95]^, platelet^[96]^, and cancer cells^[97]^) and bacterial cells^[40,98]^, have been used for membrane sources. The bacterial membrane could be used for vaccination because of the immunogenic caused by peptidoglycan and outer membrane proteins^[99]^. Recently, BEV-coated multi-antigenic nano-vaccines have been developed. BEV coating and indocyanine green (ICG)-loaded magnetic mesoporous silica nanoparticles (MSN) were developed by Chen *et al.*^[80] ^to regulate antigen presentation pathways in dendritic cells. The *in vivo *data show that the BEV-ICG-MSNs vaccine could target lymph nodes from the injection site^[80]^. 

### Biological engineering

We can use biological engineering to fuse the gene sequence of the protein with the gene sequence of the selected membrane protein^[75]^. The most commonly used methods of biological engineering are LAMP-2B [Figure 3C]^[71,83,85,100,101] ^and CD63 [Figure 3D]^[86,87,102]^. LAMP-2B, a member of the lysosome-associated membrane protein family, is the most widely used MEV membrane protein for displaying targeting motifs. The N-terminus of LAMP-2B is present on the outer surface of MEVs, and any targeting sequences can be added [Figure 3B]. Alvarez-Erviti *et al.*^[83] ^used EVs derived from dendritic cells containing Lamp2B-RVG (neuron-specific rabies viral glycoprotein) to achieve neuronal cell (Neuro-2a) targeting. To obtain colorectal tumor (HCT-116) targeting abilities, Liang *et al.*^[84] ^fused a human epidermal growth factor receptor 2 (HER2) affibody to the N-terminus of LAMP-2B. Xu *et al.*^[71] ^fused peptide E7 and LAMP-2B to produce MEVs with the ability to target synovial fluid-derived MSCs. By fusing a chondrocyte-affinity peptide with LAMP-2B, Liang *et al.*^[85] ^generated chondrocyte-targeting MEVs. The transmembrane protein CD63 can also be used to display targeting sequences [Figure 3C]. Engineered MEVs with hepatocellular carcinoma (HepG2)-targeting ability were developed by expression in 293T cell hosts and gene fusion between the CD63 and ApoA-1 sequences^[86]^. For targeting CD8^+ ^T cells, Kanuma *et al.*^[87] ^constructed engineered MEVs by fusing ovalbumin (OVA) antigen to CD63.

### Chemical engineering

Chemical engineering is another common method that allows various ligands to be displayed in the membrane by covalent reactions [Figure 3E]^[103-105] ^or noncovalent reactions [Figure 3F]^[106-108]^. The most applied covalent reactions include click chemistry bioconjugation and aldehyde amine condensation. Click chemistry has been the most used method for attaching targeting peptides to the surface of MEVs in recent years^[12,88,109]^. Sulfhydryl groups, widely present in membrane proteins, can react with maleimide by Michael addition reaction, which is usually used to selectively modify protein sites. Therefore, various functional molecules are added to the surface of MEVs by conjugating sulfhydryl groups. Jia *et al.*^[88] ^and Tian *et al.*^[12] ^also applied click chemistry to develop targeting MEVs, which have glioma-targeting and cerebral vascular endothelial cell-targeting abilities, respectively. In addition, the binding of the anchor peptides CP05 and CD63 via covalent bonding is an example of bioconjugation, indicating that engineered MEVs have emerging prospects in targeted therapy^[103]^. Tran *et al.*^[16] ^reported that the combination of aptamers and molecularly targeted MEVs is an intelligent engineering nanovesicle for precision medicine. Moreover, MEVs could be conjugated to aptamers by N-ethyl-N’-[3-(dimethylamino) propyl] carbodiimide/N-hydroxysuccinimide amidation and aldehyde amine condensation reactions^[82]^.

The most commonly applied noncovalent reactions include hydrophobic insertion, multivalent electrostatic interactions, and receptor-ligand binding [Figure 3E]. Lipids or amphiphilic molecules can be inserted into the lipid bilayer of MEVs, and the hydrophilic part is displayed on the exterior. The hydrophobic insertion can be easily accomplished with cells and MEVs at different temperatures. The commercial amphiphilic molecule DSPE-PEG can couple with ligands such as aminoethylanisamide (AA)^[110]^, RGD^[107,111]^, folate^[112]^, *etc.*, to enhance the targeting ability of MEVs. Multivalent electrostatic interactions and receptor-ligand binding are less frequently applied to MEVs. Nakase *et al.*^[113] ^utilized the negatively charged characteristics of MEVs to bind cationic lipids, which promoted the formation of pH-sensitive fusion peptides and MEVs. Qi *et al.*^[89,90] ^constructed targeted and magnetic MEVs by receptor-ligand binding. These researchers coincubated reticulocyte-derived EVs (containing transferrin receptors on the membrane surface) and transferrin-conjugated multiple superparamagnetic iron oxide nanoparticles. Importantly, Gujrati *et al.*^[20] ^used this method to construct engineered BEVs with an anti-HER2 affibody on the outer membrane surface. The engineered BEVs could target and kill cancer cells without nonspecific side effects. Although research on BEVs is not as developed as that on MEVs, their membrane structures are similar. Therefore, the engineering techniques of MEVs provide a good foundation for the in-depth study of BEVs in the future.

## THE APPLICATION OF ENGINEERED MEVS AND BEVS IN TARGETED THERAPY

**Figure 4:**
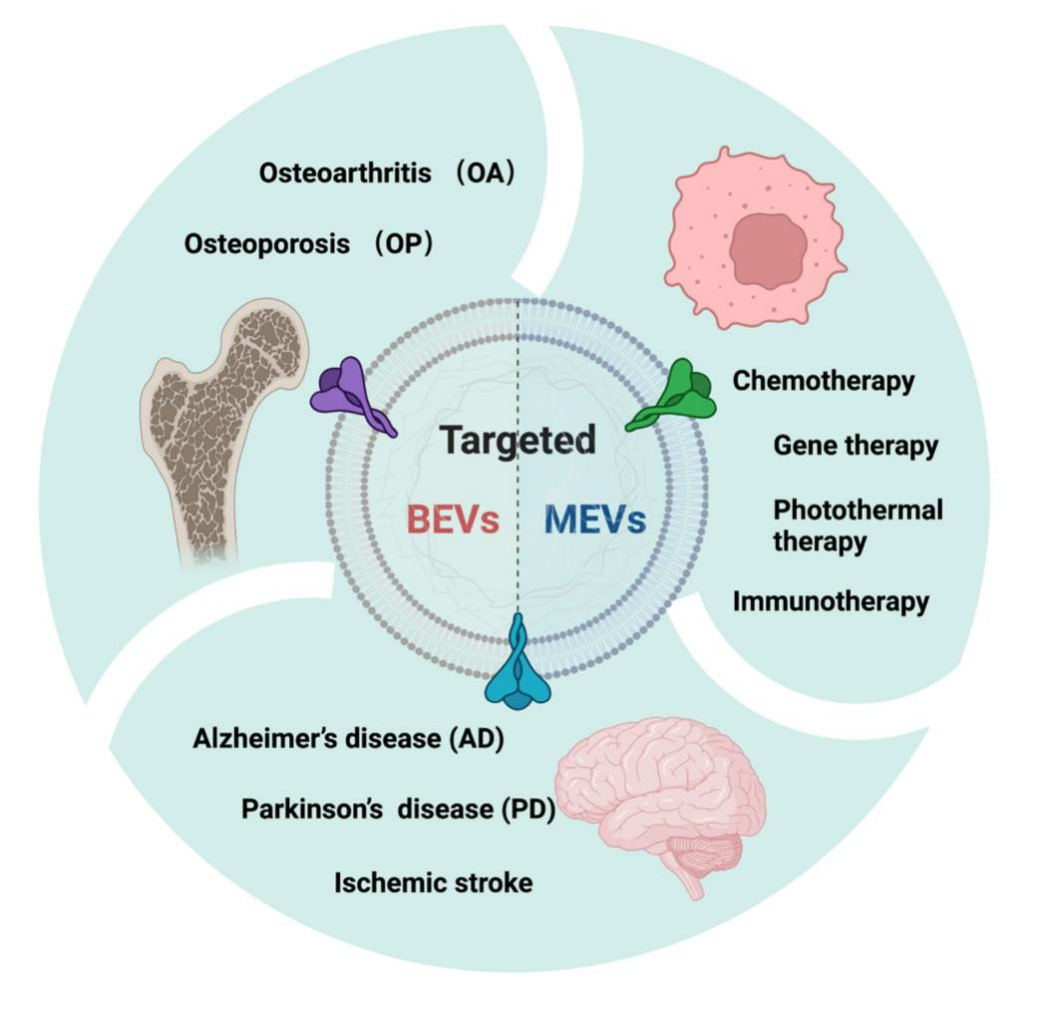
The application of engineered MEVs and BEVs in targeted therapy. The figure was created with Biorender.com. MEVs: Mammalian extracellular vesicles; BEVs: bacterial extracellular vesicles.

We summarize above the biogenesis, isolation, and characterization methods of MEVs and BEVs, as well as the various engineering methods, and engineered MEVs and BEVs can be used for targeted therapy in a variety of tissues. Next, we summarize the applications of engineered MEVs and BEVs with an enhanced targeting ability in tumors, brain, and bone diseases [Figure 4 and Table 2]. 

**Table 2:** Summary of the application of engineered MEVs and BEVs in cancer, brain, and bone disease

**Disease/Therapy**	**EV source**	**Ref.**
**Cancer**		
Chemotherapy	Mouse immature DCs Serum MDA231/B16F10 cells	^[100]^ ^[89]^ ^[114]^
Gene therapy	HEK293T Mouse 4 T1 cells *E. coli* K-12 W3110	^[101]^ ^[115]^ ^[20]^
Photothermal therapy	4T1/SKBR3/HepG2 cells MCF-7 cells *E. coli* K-12 W3110	^[116]^ ^[111]^ ^[15,117]^
Immunotherapy	CAR-T cells HEK-293T cells	^[118]^ ^[119]^
**Brain**		
Alzheimer’s disease Parkinson’s disease Ischemic stroke	Dendritic cells Mesenchymal stem cells Dendritic cells HEK293T cells HEK293T cells HEK293T Mesenchymal stem cells	^[83]^ ^[120]^ ^[121]^ ^[81]^ ^[122]^ ^[123]^ ^[124]^
**Bone**		
Osteoarthritis Osteoporosis	Mesenchymal stem cells Chondrocyte Endothelial cells NIH-3T3 cells.	^[71]^ ^[85]^ ^[17]^ ^[18]^

MEVs: Mammalian extracellular vesicles; BEVs: bacterial extracellular vesicles; EV: extracellular vesicles.

### Cancer

Various natural nanoscale MEVs and BEVs have been applied as drug delivery nanocarriers in cancer therapy^[125-129]^. The engineered targeting MEVs and BEVs show enhanced therapeutic effects for future cancer therapy, including chemotherapy, gene therapy, photothermal therapy, and immunotherapy.

#### Chemotherapy

**Figure 5:**
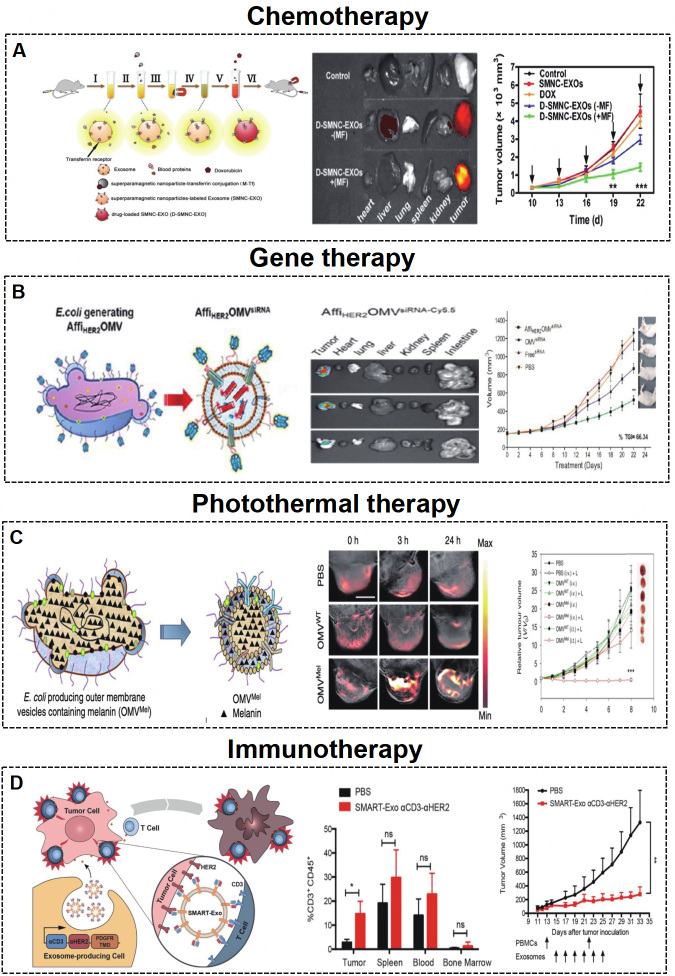
(A) Engineered MEVs for tumor chemotherapy. Schematic illustration of the construction and delivery of doxorubicin loaded in MEVs, which show tumor targeting and antitumor effects^[89]^. Copyright 2016 American Chemical Society. (B) Engineered BEVs for tumor gene therapy. Schematic illustration of the construction and delivery of siRNA loaded in BEVs, which show tumor targeting and antitumor effects^[20]^. Copyright 2014 American Chemical Society. (C) Engineered MEVs for tumor photothermal therapy^[15]^. Schematic illustration of the construction of BEV-Mel, which shows tumor targeting and antitumor effects. Copyright 2019 Springer Nature. (D) Engineered MEVs for tumor immunotherapy^[119]^. Schematic illustration of the construction of SMART-MEVs, which show tumor targeting and antitumor effects. Copyright 2020 Elsevier. MEVs: Mammalian extracellular vesicles; BEVs: bacterial extracellular vesicles; SMART-MEVs: synthetic multivalent antibodies retargeted MEVs. Significance of finding was defined as follows: not significant, ^ns^*P *> 0.05; ^*^P < 0.05; ^**^P < 0.01; ^***^P < 0.001.

The treatment of tumor diseases routinely involves chemotherapeutic drugs, but chemotherapy drugs do not show specific targeting and have significant cytotoxic side effects, resulting in poor therapeutic effects. Targeted drug delivery based on engineered MEVs and BEVs could increase the local concentration and minimize cytotoxic side effects, consequently improving efficacy. Tian *et al.*^[100] ^constructed iRGD-MEVs for the delivery of doxorubicin to the breast cancer cell line MDA-MB-231. The chemotherapeutic drug doxorubicin was encapsulated in targeting iRGD-MEVs by electroporation technology. Intravenous injection of iRGD-MEVs specifically delivered doxorubicin to tumor tissues and resulted in inhibition of tumor growth. Qi *et al.*^[89] ^developed dual-functional (magnetic and targeting ability) MEVs loaded with doxorubicin to target hepatoma 22 subcutaneous cancer cells. Dual-functional MEVs enhanced the cancer-targeting ability under a magnetic ﬁeld and suppressed tumor growth [Figure 5A]. Similarly, A33 antibody functionalized MEVs with doxorubicin were used to target the colorectal cancer cell line LIM1215^[114]^.

#### Gene therapy

Gene therapy is a strategy to correct or compensate for abnormal gene expression in tumor cells by delivering nucleic acids such as siRNAs, miRNAs, *etc.*, to achieve the purpose of treatment, which has proven to be a promising cancer treatment approach^[130]^. Specifically, EVs can protect RNA from degradation, which ensures the stability and bioactivity of RNA after targeting cells^[131]^. Bai *et al.*^[101] ^reported engineered targeting tLyp-1 MEVs for efficient delivery of *SOX2 *siRNA to HEK293T cells. The engineered tLyp-1 MEVs had high transfection efficiency in non-small-cell lung cancer (NSCLC) and a high *SOX2 *gene silencing ability in NSCLC stem cells. Zhao *et al.*^[115] ^exploited biomimetic CBSA-MEV nanoparticles loaded with S100A4 siRNA, which effectively targeted the lung and showed excellent gene-silencing eﬀects. Moreover, bioengineered BEVs have been used for targeted therapy of tumors. Gujrati *et al.*^[20] ^constructed BEVs with low immunogenicity that can target cancer cells by delivering KSP siRNA [Figure 5B].

#### Photothermal therapy

**Figure 6:**
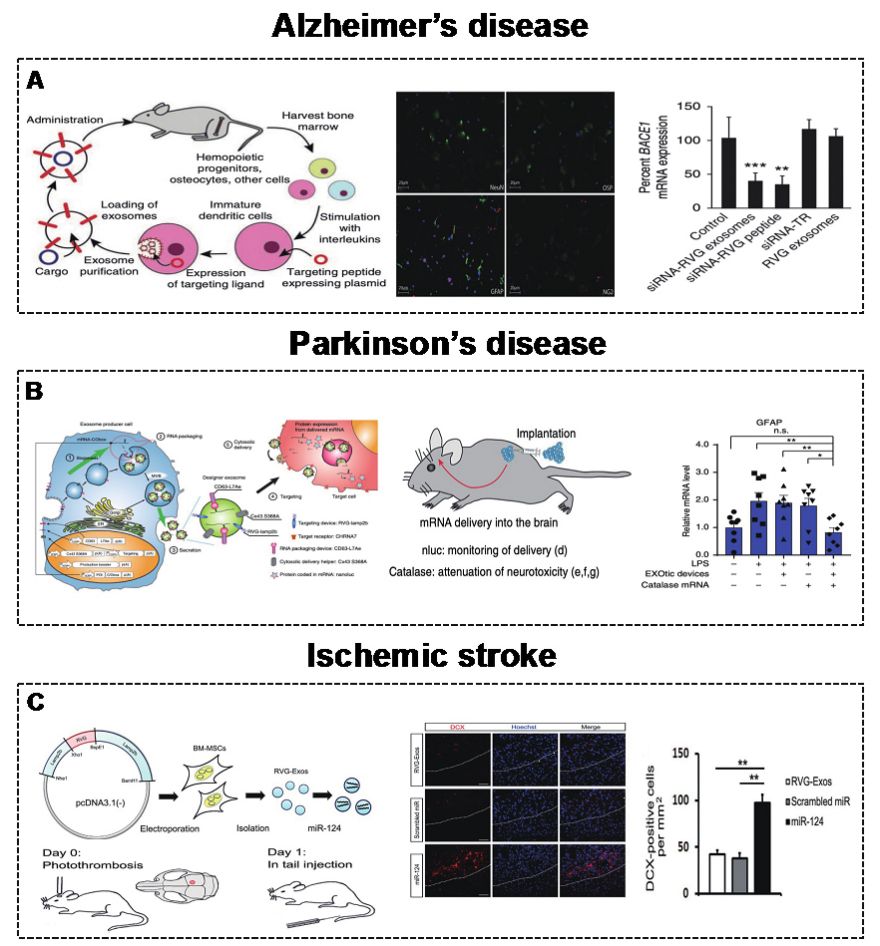
(A) Engineered MEVs for AD. Schematic illustration of the construction of MEVs to deliver BACE1 mRNA with targeting and anti-AD effects^[83]^. Copyright 2011 Springer Nature. (B) Engineered MEVs for PD. Schematic illustration of the construction of MEVs to deliver catalase mRNA with targeting and anti-PD effects^[81]^. Copyright 2018 Springer Nature. (C) Engineered MEVs for ischemic stroke.Schematic illustration of the construction of MEVs to deliver catalase miR-124 with targeting and anti-ischemic stroke effects^[124]^. Copyright 2017 Elsevier. MEVs: Mammalian extracellular vesicles. AD: Alzheimer’s disease. PD: Parkinson’s disease. Significance of finding was defined as follows: not significant, ^ns^*P *> 0.05; ^*^P < 0.05; ^**^P < 0.01; ^***^P < 0.001.

The problems of recurrence, drug toxicity, and multidrug resistance are still difficult to overcome with traditional surgical intervention, chemotherapy, and gene therapy. Photothermal therapy is a nontoxic and noninvasive tumor-targeted treatment method^[111,116,132,133]^. The combination of engineered bioactive material loaded EVs and photothermal therapy is a promising method for cancer therapy. Bose *et al.*^[116] ^developed MEVs loaded with anti-miRNA-21-coated gold-iron oxide nanoparticles (GIONs). MEV-GIONs showed strong T2 contrast in magnetic resonance imaging and photothermal effects in breast cancer 4T1 cells. Cao *et al.*^[111] ^constructed Arg-Gly-Asp (RGD) peptide-MEVs coated with vanadium carbide quantum dots. The resulting MEVs could target cancer cells and access the nucleus to induce low-temperature photothermal therapy, which showed effective tumor destruction. In addition, Mel is highly suitable for photothermal therapy due to its good photothermal conversion efficiency^[134,135]^. Gujrati *et al.*^[15,117] ^introduced engineered BEVs derived from *E. coli *W3110△*msbB *to carry Mel [Figure 6C]. The engineered BEV-Mel is an excellent anticancer therapy due to its targeting ability, biocompatibility, and scalability. Importantly, BEV-Mel did not induce chronic systemic toxicity or side effects [Figure 5C].

#### Immunotherapy

Immunotherapy is another promising method in the field of tumor therapy^[119,136]^. Precision targeted therapy with chimeric antigen receptor T (CAR-T) cells is a new type of tumor treatment that has achieved good results in clinical tumor treatment. Fu *et al.*^[118] ^introduced engineered MEVs derived from CAR-T cells. CAR-containing MEVs could express many cytotoxic molecules and target and kill cancer cells. Shi *et al.*^[119] ^developed synthetic multivalent antibodies retargeted MEVs (SMART-MEVs), which could specifically target CD3-positive T cells and HER-2 breast cancer cells. The SMART-MEVs exhibited valid and specific antitumor effects [Figure 5D].

### Brain 

In addition to their strong loading capacity, low toxicity, and low immunogenicity, EVs can also cross the blood-brain barrier. Therefore, EVs can be widely used as a therapeutic vehicle for brain and neurodegenerative diseases^[13,137,138]^. Here, we summarize the therapeutic effects of engineered targeting EVs in brain diseases, such as AD, PD, and ischemic stroke.

#### Alzheimer’s disease 

AD is a progressive neurodegenerative disease involving the superfluous accumulation of β-amyloid, which is produced by the BACE1 protein^[139]^. Therefore, controlling the expression of the BACE1 protein is an effective way to control AD. Alvarez-Erviti *et al.*^[83] ^described engineered RVG (central nervous system-specific peptide)-MEVs (derived from DCs) to specifically deliver GAPDH siRNA to neurons, oligodendrocytes, and microglia in the brain. Mice were injected intravenously with RVG-MEVs, and the engineered MEVs resulted in a significant decrease in the expression of BACE1 mRNA and protein [Figure 6A]. Cui *et al.*^[120] ^also demonstrated that intravenously infused RVG-MEVs (derived from MSCs) show strong targeting to the cortex and hippocampus, effectively improving learning and memory abilities. 

#### Parkinson’s disease

PD is another progressive neurodegenerative disease that involves the formation of Lewy bodies, which is affected by excessive accumulation of α-synuclein (α-Syn)^[140-142]^. Similarly, decreasing α-synuclein in brain cells could delay PD. Cooper *et al.*^[121] ^delivered α-Syn siRNA by RVG-MEVs (derived from murine dendritic cells) to reduce α-Syn accumulation in the brain. Kojima *et al.*^[81] ^developed MEVs with targeting, cytoplasmic delivery capabilities, and specific RNA encapsulation by EV production booster devices. The delivery of therapeutic catalase mRNA significantly alleviated neurotoxicity and neuroinflammation in mice [Figure 6B]. Liu *et al.*^[122] ^also modified the membrane surface with the RVG peptide for the targeting ability of MEVs, which delivered MOR siRNA to Neuro2A cells in the brain, leading to decreased morphine addiction.

#### Ischemic stroke

Ischemic stroke is a disease caused by cerebral arterial stenosis that releases high-mobility group box 1 (HMGB1) to the extracellular spaces and results in inflammatory reactions^[143]^. The knockdown of HMGB1 in the brain may be an effective anti-inflammatory strategy to improve ischemic stroke. Kim^[123] ^applied brain-targeting RVG-MEVs (derived from HEK293T cells) to precisely deliver HMGB1 siRNA. HMGB1 siRNA was loaded by electroporation technology. Engineered RVG-MEVs with HMGB1 siRNA successfully reduced the expression of HMGB1 protein and apoptosis levels in the brain. In addition, the delivery of miRNAs such as miR-124 is involved in the neuro-remodeling process^[144,145]^. Using this strategy, Yang *et al.*^[124] ^constructed RVG-MEVs to deliver miR-124 to the infarct site and protect against ischemic injury [Figure 6C].

### Bone

Bone is an internal support system that provides the structural foundation for the human body and muscle^[146-149]^. The most common bone diseases, such as OA, OP, bone fractures, and bone defects, have been linked to MEVs and BEVs^[150-153]^. The application of engineering techniques to enhance the bone targeting ability of EVs has substantially increased their therapeutic efficacy in these bone-related diseases. Conventional fractures or bone defects often require biomaterials, such as hydrogels^[154-156] ^and scaffolds^[157-160]^, for therapeutic effects. Here, we review the application of targeted EVs in OA and OP.

#### Osteoarthritis 

**Figure 7:**
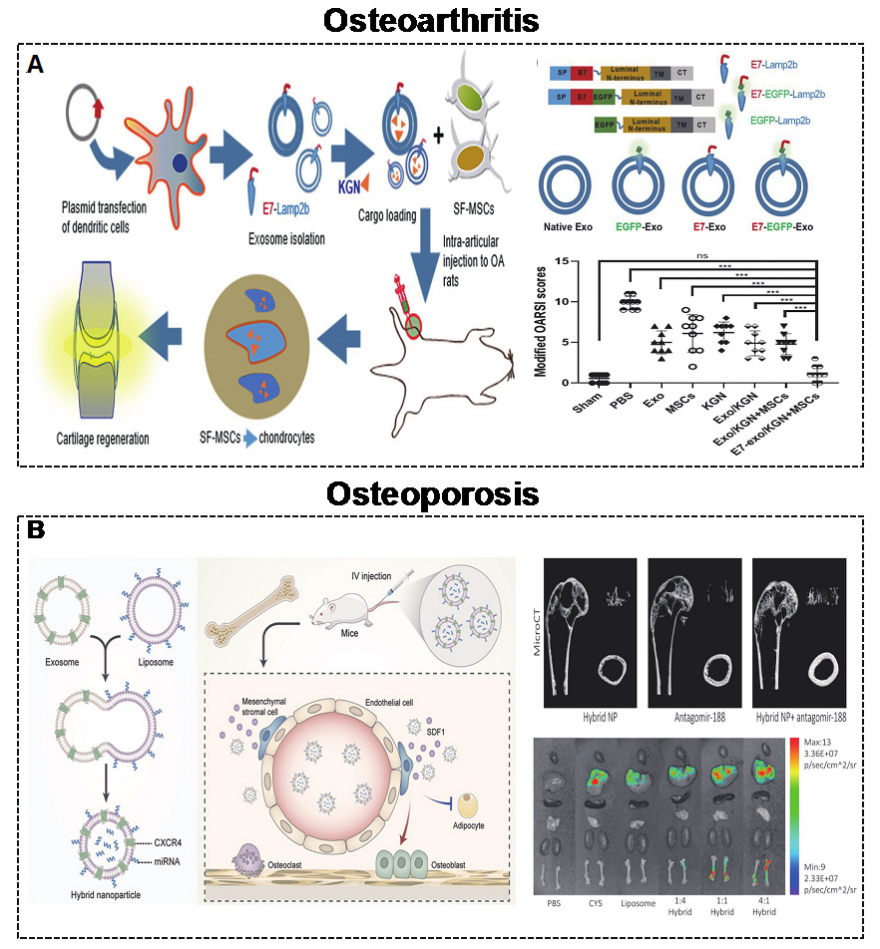
(A) Engineered MEVs for OA. Schematic illustration of the construction of MEVs to deliver KGN with targeting and anti-OA effects^[71]^. Copyright 2021 Springer Nature. (B) Engineered MEVs for OP. Schematic illustration of the construction of hybrid nanoparticles to deliver antagomir-188 with targeting and anti-OP effects^[18]^. Copyright 2021 Elsevier. MEVs: Mammalian extracellular vesicles; OA: osteoarthritis; KGN: kartogenin; OP: osteoporosis. Significance of finding was defined as follows: not significant, ^ns^*P *> 0.05; ***P < 0.0001.

OA is a common joint disease with no recognized mechanism^[161]^. Cartilage degeneration, subchondral bone sclerosis, and synovial inﬂammation are prominent features of OA^[162,163]^. There are no effective OA treatments approved by official agencies, except for joint replacement. Although the precise mechanism of OA is still unclear, EVs, especially targeted EVs, play a vital role during the progression of OA, indicating their exciting therapeutic prospects^[164]^. The small molecule drug kartogenin (KGN) was shown to induce synovial fluid-derived MSCs (SF-MSCs) to differentiate into chondrocytes^[165-167]^. Xu *et al.*^[71] ^reported the targeted delivery of KGN to SF-MSCs by engineered MEVs to accelerate chondrogenesis [Figure 7A]. The targeting ability of engineered MEVs is due to E7-Lamp2B. KGN was loaded inside MEVs by electroporation. Moreover, miR-140 is regarded as a promising agent for the treatment of OA due to its dual roles in both homeostasis and cartilage^[168,169]^. Liang *et al.*^[85] ^reported a similar targeted strategy to deliver miR-140 to chondrocytes by engineered MEVs to alleviate the progression of OA. The targeting ability of engineered MEVs is due to CAP-Lamp2B. miR-140 was also introduced into MEVs by electroporation.

#### Osteoporosis 

OP is a systemic metabolic disease of the skeletal system characterized by fragility fracture^[170-172]^. The main cause of OP is an imbalance in the metabolism of osteoblasts and osteoclasts. Postmenopausal women suffer from osteoporosis-related fractures throughout their lifetime^[173]^. Bone-targeted EVs are optimal interventions to improve postmenopausal OP (PMO). Our group demonstrated that MEVs derived from endothelial cells have a better targeting ability than those from osteoblasts^[17]^. In addition, our team developed targeting MEVs by displaying C-X-C motif chemokine receptor 4 (CXCR4) on their surface^[18]^. miR-188 was shown to inhibit osteogenesis of bone marrow mesenchymal stem cells (BMSCs), and the knockdown of miR-188 also improved bone loss^[174]^. Therefore, we combined CXCR4^+ ^with liposomes containing antagomir-188 to generate hybrid nanoparticles for OP treatment [Figure 7B].

## CONCLUSION AND PERSPECTIVE

Over time, the pivotal role of EVs in cell-to-cell communications, in contrast to their initial roles as “garbage bags”, has been established. Nanosized EVs have many advantages, such as rich functional contents, a stable membrane structure, good biocompatibility, low immunogenicity, *etc. *Such cell membrane-derived vesicles have been explored in prokaryotic and eukaryotic cells^[95,175,176]^. As the two most common types of EVs in the biomedical field, MEVs and BEVs have been studied, resulting in major progress in the biogenesis, isolation, and characterization of these vesicles. For biogenesis, MEVs are formed by endosomal systems including sequential plasma membrane invagination and membrane fusion of eukaryotic cells. BEVs are generated by endolysin-triggered cell lysis and membrane blebbing of bacteria. The isolation and characterization of MEVs and BEVs determine their further application in biomedicine. Here, an ultracentrifugation-based isolation protocol is described for MEVs. An effective isolation method based on ultracentrifugation and density gradient centrifugation is also described for BEVs. For characterization, TEM and NTA are commonly used to characterize the sizes, shapes, and concentrations of these vesicles. However, different types of MEVs contain the same protein molecules, such as TSG101, CD63, and CD81, which are always used as specific markers by WB. In contrast, although several membrane proteins, such as OmpF and OmpA, have been used for the characterization of *E. coli *EVs, specific markers of BEVs are still a major challenge. 

The use of MEVs and BEVs also has many challenges, such as poor targeting specificity. Targeted drug delivery of EVs was proposed in 2011 and has since received increasing attention due to their excellent characteristics^[177]^. Targeted modification methods have been applied in MEVs and BEVs to increase the targeting ability and healing efficacy. Targeted engineering aims to increase the local concentration of EVs at diseased sites, thereby reducing toxicity and side effects and maximizing healing efficacy. Both MEVs and BEVs are lipid bilayer-enclosed structures containing various biomolecules. Therefore, most engineering methods are universal. Here, physical, biological, and chemical engineering methods based on membranes to modify MEVs are described, which could also guide the modification of BEVs. Furthermore, the applications of engineered MEVs and BEVs in targeted therapy, such as therapy for tumors and brain and bone diseases, are summarized. MEVs and BEVs with targeting capabilities are usually administered systemically intravenously, and they will accumulate at the target site for better therapeutic efficiency. In addition to the direct injection, the incorporation of MEVs and BEVs with biomaterials is also a promising approach to enhance the healing efficacy^[160]^. Different types of biomaterials, such as hydrogels^[154,178-181] ^and scaffolds^[157,159,182,183]^, have been developed to achieve high retention rates of EVs and healing efficacy of tissue. In situ injection is another targeted technique, allowing MEVs and BEVs to act directly at the injured tissues. However, some hard-to-reach tissues still require targeted EVs. The many big deals recently by large pharmaceutical companies indicate that the industry expects MEVs and BEVs to deliver drugs to hard-to-reach tissues^[184]^.

Although MEVs have been more extensively studied than BEVs, one of the challenges of MEVs is the limited yield. BEVs are easily available due to the rapid proliferative abilities, mature culture methods, and gene editing techniques of bacteria^[185,186]^. In addition, the scalability, low cost, and environmental friendliness of bacterial fermentation culture indicate that the industrialization of BEVs is possible^[187,188]^. Synthetic biology can also be used to confer additional functions on bacteria and their associated BEVs^[189,190]^. Moreover, several biotherapeutic bacteria, especially human commensal bacteria, such as *E. coli *Nissle 1917^[48]^, *A. muciniphila*^[191]^, and *L. rhamnosus *GG^[192]^, are being investigated in clinical trials^[193,194]^. Therefore, BEVs derived from probiotics are promising pharmaceutical agents in the biomedical field. Importantly, BEVs are safe because they are cell-free. Both oral and intravenous BEVs were well tolerated and resulted in low immunogenic responses^[15,19,20]^. Therefore, the topic of “nonmammalian EVs, especially BEVs” is receiving more attention in the latest MISEV. The mature application system of MEVs can also lay a solid foundation for BEVs in biomedical fields. Studies on MEVs and BEVs can inspire each other and draw important elements from each type to enhance functional and therapeutic effcacy. Overall, the rise of targeted therapeutics of engineered MEVs and BEVs shows promise for future clinical translation of EVs.

## DECLARATIONS

### Copyright

© The Author(s) 2022.
